# EBV Vaccines in the Prevention and Treatment of Nasopharyngeal Carcinoma

**DOI:** 10.3390/vaccines13050478

**Published:** 2025-04-29

**Authors:** Weiwei Zhang, Chuang Wang, Yousheng Meng, Lang He, Mingqing Dong

**Affiliations:** 1 Department of Oncology, Cancer Prevention and Treatment Institute of Chengdu, Chengdu Fifth People’s Hospital/The Second Clinical Medical College, Affiliated Fifth People’s Hospital of Chengdu University of Traditional Chinese Medicine, Chengdu 611130, China; zwwei89@126.com (W.Z.);; 2 Chengdu Yunce Medical Biotechnology Co., Ltd., Chengdu 611135, China; wangchuang@yuncetech.com; 3 Division of Pulmonary Medicine, the First Affiliated Hospital, Wenzhou Medical University, Wenzhou Key Laboratory of Interdiscipline and Translational Medicine, Wenzhou Key Laboratory of Heart and Lung, Wenzhou 325000, China

**Keywords:** Epstein–Barr virus, nasopharyngeal carcinoma, vaccines, prevent, treatment

## Abstract

Epstein–Barr virus (EBV), a ubiquitous human herpesvirus, has been robustly linked to the pathogenesis of nasopharyngeal carcinoma (NPC). The mechanism of EBV-induced NPC involves complex interactions between viral proteins and host cell pathways. This review aims to comprehensively outline the mechanism of EBV-induced NPC and the latest advances in targeted EBV vaccines for prophylaxis and treatment. This review explores the intricate molecular mechanisms by which EBV contributes to NPC pathogenesis, highlighting viral latency, genetic and epigenetic alterations, and immune evasion strategies. It emphasizes the pivotal role of key viral proteins, including EBNA1, LMP1, and LMP2A, in carcinogenesis. Subsequently, the discussion shifts towards the development of targeted EBV vaccines, including preventive vaccines aimed at preventing primary EBV infection and therapeutic vaccines aimed at treating diagnosed EBV-related NPC. The review underscores the challenges and future directions in the field, stressing the importance of developing innovative vaccine strategies and combination therapies to improve efficacy. This review synthesizes current insights into the molecular mechanisms of EBV-induced NPC and the development of EBV-targeted vaccines, highlighting the potential use of mRNA vaccines for NPC treatment.

## 1. Background

EBV infections pose a global health issue, leading to infectious mononucleosis (IM) in 70% of adolescents and adults in developed nations [1]. EBV-related cancers constitute 1.5% of global cancer cases and 1.8% of cancer-related deaths [1,2,3]. EBV is linked to various malignancies, such as nasopharyngeal carcinoma (NPC), Hodgkin’s lymphoma, Burkitt’s lymphoma, and gastric cancer [4,5,6,7]. There are more than 130,000 new cases of NPC worldwide every year [8,9]. NPC patients have extremely skewed ethnic and geographic distributions, mainly concentrated in Southeast Asia, and its incidence is usually 30 times that of Europe and America [10]. Due to the special position of nasopharynx in the human body and the untypical early symptoms of NPC, about 70% of the patients were in stage III or IV (middle and late stage) when diagnosed [11]. Simple radiotherapy or concurrent radiotherapy and chemotherapy is still an important treatment for NPC [11]. With the development of radiotherapy technology and the continuous improvement of chemotherapy regimens, the 5-year survival rate of early NPC patients undergoing radiotherapy and chemotherapy can reach more than 90% [12]. The 5-year survival rate of locally advanced NPC is about 74.5~80.0% [13,14]. Despite NPC being sensitive to chemoradiotherapy, also approximately 15–30% of NPC patients experience recurrence or distant metastasis, with a median survival of 19–21 months for those with distant metastasis [15,16], highlighting the urgent need for novel therapeutic and preventive strategies.

EBV infection is detected in nearly all cases of non-keratinizing NPC, the most common histological subtype, underscoring its critical role in the pathogenesis of this disease. EBV primarily promotes the development and progression of NPC through mechanisms such as viral latent infection, genetic and epigenetic alterations, and immune evasion. The EBV associated with NPC is usually a latent type II infection mode, and the tumor cells mainly express three latent membrane proteins (LMP), namely LMP1, LMP2A, and LMP2B. Moreover, Epstein–Barr nuclear antigen (EBNA) 1 has the capacity to trigger the transformation of epithelial cells into NPC cells and is implicated in the processes of tumor invasion and metastasis [17].

EBV vaccines have emerged as a promising approach to combat EBV-associated malignancies, including NPC. Prophylactic vaccines aim to prevent primary EBV infection, while therapeutic vaccines are designed to elicit robust immune responses against EBV-infected or transformed cells, thereby controlling tumor progression. Unfortunately, up to now, no vaccine targeting EBV has been approved in the world. Recent advancements in vaccine technology, such as mRNA-based platforms and multivalent antigen designs, have revitalized interest in EBV vaccine development. These innovations offer the potential to enhance immunogenicity and target multiple EBV antigens simultaneously, addressing the challenges posed by viral latency and immune evasion. Based on the reported studies, this paper systematically summarized the relevant mechanisms of EBV infection and NPC, and further discussed the development of EBV vaccines in prevention and treatment of NPC.

## 2. Characteristics and Infection Patterns of EBV

EBV, a member of the gamma–herpesvirus subfamily, is the sole virus within this group known to infect humans. It was first discovered in 1964 and named after its discoverer [18]. EBV enters the human body mainly through contact with body fluids [19]. EBV comprises approximately 170 kb of linear double-stranded DNA, encoding over 80 proteins and 46 untranslated functional small RNAs [20,21]. EBV isolates are classified into type 1 and type 2 based on the sequence of the EBNA gene [22]. Type 1 EBV is prevalent in Asian and Western populations and is the most common subtype associated with NPC. Type 2 EBV is primarily found in African regions. Compared to type 2, type 1 EBV induces a stronger transformation of B cells into lymphoblastoid cell lines. Unlike EBV strains originating from IM and Burkitt’s lymphoma, the EBV strain isolated from NPC demonstrates epithelial tropism and exhibits enhanced lytic activity upon infecting B cells.

The EBV life cycle, like other herpesviruses, includes two main phases: the incubation period and the cleavage period. Latency is a crucial characteristic of EBV, as the virus maintains a lifelong presence in the human body, with only occasional reactivation and lytic replication. EBV in incubation periods does not produce new virions and has less gene expression. The expression of viral genes during EBV latent infection varies with the cellular environment, and the EBV fragment undergoes rolling cycle replication to form a linearized genome. The EBV linear genome enters the nucleus, where it circularizes through terminal repeats, forming stable episomes that establish latent infection [23]. Following EBV infection, nine proteins are expressed during replicative infection: six viral nuclear antigens (EBNA1, EBNA2, EBNA3A, EBNA3B, EBNA3C, and EBNA- leader protein (LP)) and three LMPs (LMP1, LMP2A, and LMP2B). The latent period can be categorized into three distinct modes: type I, type II, and type III. All three modes exhibit expression of EBV-encoded small RNAs (EBER) and EBNA1 [24]. EBERs are abundantly expressed in all EBV latency types and promote cell survival by inhibiting PKR-mediated antiviral responses [25]. BamHI-A region rightward transcript (BART) miRNAs show differential expression: highly upregulated in Latency I/II (e.g., NPC) to target tumor suppressors (e.g., PTEN), while weakly expressed in Latency III [26,27]. The latent period EBV can permanently exist in memory B cells [28,29]. Type I latency is only expressed by EBNA1 and is present in Burkitt lymphoma [20]. Type II latency mainly exists in Hodgkin lymphoma, NPC, and gastric cancer, expressing LMP1, LMP2, and EBNA1 [30]. The type III latent period is mainly seen in post-transplant lymphoproliferative diseases and immune cell lymphoma of HIV-infected individuals. At this time, all eight latent antigens of EBV are expressed, including EBNA1, EBNA2, EBNA3 family (3A/3B/3C), EBNA-LP, LMP1, and LMP2 [8]. In addition, type II latent infection is the main infection mode of NPC, which is characterized by EBER-positive detection in tumor tissue and EBV-related antigen expression [31].

The lytic phase involves ongoing virion production and the persistent breakdown of infected cells. In this process, EBV regulates the production of new virions and induces immune escape through the expression of more than 80 lytic proteins [32,33]. These lytic proteins systematically disrupt host defense through three key mechanisms: (1) antigen presentation blockade—BGLF5 degrades major histocompatibility complex (MHC)-I/II mRNAs while BILF1 internalizes surface MHC-I, crippling T cell recognition [34,35]; (2) innate immunity suppression—BNRF1 inhibits STING/TBK1 signaling to disrupt interferon responses [36,37] and BRLF1 inhibits transcription of IRF3 and IRF7 and suppresses induction of IFN-β [38]; (3) microenvironment manipulation—viral Bcl-2 proteins (vBcl-2s) protect the virus from apoptosis in its host cell during virus synthesis [39], while vIL-10 (BCRF1) mimics human IL-10 to suppress pro-inflammatory cytokines [40]. Additionally, gp42 sequesters MHC-II to impair CD4+ T cell help [41], and BARF1 binds colony-stimulating factor-1 to disrupt myeloid cell functions. This multilayered strategy enables EBV to produce progeny virions despite immune surveillance, facilitating viral transmission and persistence.

The cleavage stage, following EBV entry, can be subdivided into early, middle, and late phases based on distinct gene-expression profiles [42].The Rta protein from the BRLF1 gene and the Zta protein from the BamHI Z Leftward Reading Frame 1 (BZLF1) gene are transcription factors for early genes. Rta and Zta are crucial proteins for transitioning from latency to the cleavage stage. The Rta protein not only induces the lysis cycle but also stabilizes certain capsid and envelope proteins [43,44,45,46]. In addition, viral antigens, proteases, and glycoproteins are proteins required for assembly of late gene-encoded virions [47].

In NPC, EBV predominantly exists in a latent state. During this phase, the sustained expression of viral genes such as LMP1 and LMP2A promotes the transformation and proliferation of host cells, thereby establishing a foundation for NPC development [48,49,50]. Additionally, EBV-infected cells employ immune-evasion mechanisms during latency to avoid clearance by the host immune system, enabling their survival and progression into NPC [51]. During EBV lytic reactivation in NPC, infected cells release viral particles and tumor-promoting cytokines, driving the transformation and progression of surrounding tumor cells [48,52]. Furthermore, lytic-phase cells contribute to NPC pathogenesis through non-cell-autonomous mechanisms, including angiogenesis and extracellular matrix remodeling [52]. In summary, both the latent and lytic phases of EBV infection play critical roles in NPC development. Latent viral gene expression and immune evasion mechanisms underlie NPC initiation, while lytic-phase activities—including viral particle release and cytokine production—promote tumor progression [51].

## 3. Mechanism of EBV-Induced Pathogenesis of NPC

Analogous to the architecture of other herpesviruses, EBV is an enveloped virus featuring multiple viral glycoproteins, including gp350, gp42, gH, gL, and gB, which play pivotal roles in receptor recognition, viral adherence, and the fusion of virus–host membranes [53]. EBV mainly infects B lymphocytes and epithelial cells. During infection of B lymphocytes, the EBV envelopes glycoprotein gp350 and initiates the infection process by binding to the CD21 receptor on the B lymphocyte surface. CD21 serves as the primary receptor for EBV infection of B cells, facilitating viral entry through its interaction with the EBV glycoprotein gp350/220 [54]. CD21 (complement receptor 2, CR2) is the main receptor for EBV infection of B cells. The envelope glycoprotein gp350/220 of EBV specifically binds to CD21, mediating virus attachment and internalization, thereby initiating B cell infection [55,56]. CD21 not only mediates EBV invasion, but also participates in viral latent infection and immune escape. As part of the B cell co-receptor complex (CD19/CD21/CD81), CD21 enhances the BCR signaling pathway, promoting the survival and proliferation of infected B cells [57]. According to Jiang et al.’s study, the abnormal upregulated expression of CD21 in nasopharyngeal epithelial cells may be the main reason for promoting the entry of EBV into epithelial cells and inducing pathogenesis [58]. Subsequently, gp42 engages with gp350, facilitating the interaction between MHC class II and the gH/gL heterodimer. Ultimately, gH/gL activates the EBV fusion glycoprotein gB, resulting in the fusion of the EBV envelope with the B lymphocyte membrane [59,60,61].

**Figure 1:**
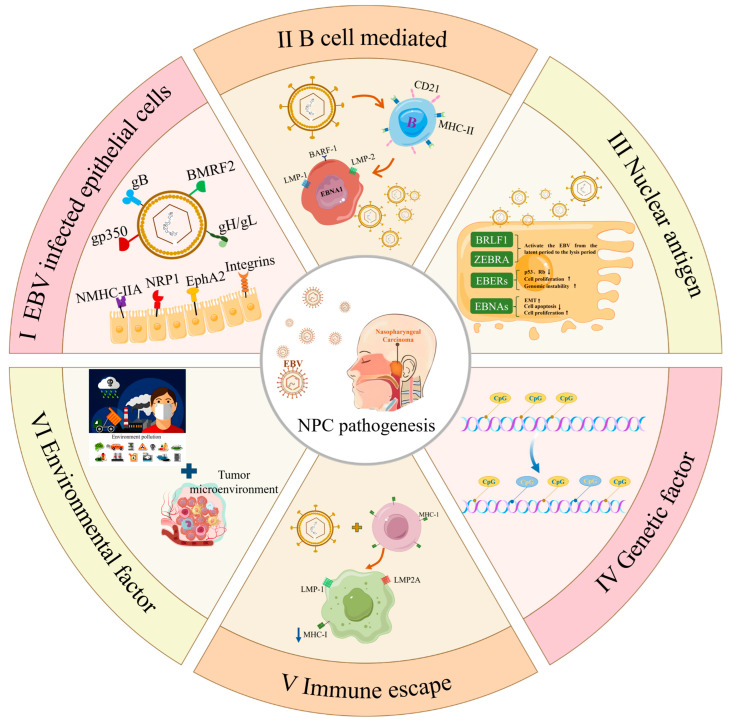
The mechanism by which EBV promotes the occurrence and development of nasopharyngeal carcinoma. (I) EBV can directly infect epithelial cells; the envelope proteins gH/gL and gB of EBV play a critical role in the process. For example, gH and gL can form heterodimers and bind to integrins αVβ5, αVβ6, or αVβ8 on the epithelial cell surface in the early stage [72,74]. And EphA2 is also a key receptor for EBV to invade epithelial cells [82]. NRP1 is an important molecule for EBV to enter nasopharyngeal epithelial cells. NRP1 can interact with the CendR special domain of EBV glycoprotein gB, enabling EBV to enter nasopharyngeal epithelial cells through macrocytic and lipid raff-dependent endocytosis mechanisms and activating the NrP1-dependent EGFR-ERK signaling pathway. The activation of the EGFR-ERK signaling pathway and the expression of NRP1 are two necessary factors for efficient EBV infection [69]. (II) EBV can efficiently transduce B cells to turn to immortalized lymphoblasts, and contact with epithelial cells to transfer viruses, and then induce NPC [83,84]. (III) EBNAs are a group of nuclear proteins encoded by EBV, which plays an important role in the pathogenesis of NPC. For example, EBNA1 can inhibit p53 and Rb, promote cell proliferation and genomic instability, and promote the proliferation and survival of tumor cells through various mechanisms [85,86,87,88].Arrow ↑ represents promotion and arrow ↓ represents inhibition. (IV) EBV infection can cause CpG upregulation, histone modification, change in proto-oncogene expression, leading to cell proliferation and apoptosis inhibition, and genome instability [89,90,91,92]. (V) The expression of LMP1 and LMP2A in EBV-infected cells downregulated MHC-I and reduced antigen presentation, leading to immune escape [93,94]. (VI) Environmental factors mainly include macro and micro aspects. Microscopic factors mainly form a favorable microenvironment for tumor growth through the interaction between viral proteins and host cytokines [95,96,97]. Macro factors mainly include environmental pollution, smoking, diet, formaldehyde, dust, heavy metal inhalation, etc. [98,99,100,101].

Additionally, several studies have also shown that when EBV infects epithelial cells, EBV glycoprotein Bone Morphogenetic Protein Receptor 2 (BMRF2) binds to the integrin on the surface of epithelial cells and then binds to gH/gL, promoting the fusion of the virus envelope and the epithelial cell plasma membrane. Finally, we promote EBV to enter epithelial cells [1,62,63,64,65,66,67,68]. Wang et al. and Xiong et al. studied two other receptors on nasopharyngeal epithelial cells, Neuropilin-1 (NRP1) and non-muscular myosin heavy chain IIA (NMHC-IIA); they are combined with EBV glycoprotein gB and EBV glycoprotein gH/gL to promote EBV into epithelial cells [69,70]. NRP1 and αvβ6/β1 integrins play distinct but complementary roles in EBV infection of epithelial cells: NRP1 binds EBV glycoprotein B (gB) to enhance viral entry and maintain latency, while integrins mediate viral attachment through gH/gL interactions and trigger PI3K/Akt-dependent internalization [71,72,73,74]. Both receptors promote immune evasion, with NRP1 inducing TGF-β signaling and integrins upregulating PD-L1 to create an immunosuppressive niche [75,76] (Figure 1). The study of Kang et al. found that the high expression of BART miRNAs may be one of the mechanisms of NPC [77]. EphA2 is believed to play a key role in the process of EBV infection of epithelial cells [78], which can directly bind EBV glycoprotein gB and gH/gL and promote EBV endocytosis and fusion [79]. Moreover, the EBD and FNR domains of EphA2 extracellular segment are indispensable for mediating EBV infection to epithelial cells to induce NPC [80,81].

At present, there are different reports on the mechanism of EBV-induced NPC. However, the exact path of EBV entering nasopharyngeal epithelial cells has not been clearly concluded up to now. From the main characteristics of herpesvirus infecting human cells, we speculate that EBV is more dependent on the combination/internalization of various glycoproteins expressed on the viral shell membrane to enter epithelial cells, proliferate after incubation and cleavage, and directly induce gene mutation or abnormal methylation of epithelial cells to cause NPC. The development and progression of NPC may be influenced by various factors, including environmental conditions and genetic mutations, due to variations in the human immune microenvironment. Further research is required to substantiate this multifactorial induction. The molecular mechanism is summarized in Figure 1.

### 3.1. EBNA1 and NPC

The glycoprotein encoded by EBV is a key factor in promoting NPC development. EBNA1, expressed in all EBV-associated tumors, is a crucial viral protein that promotes NPC and is essential for sustaining EBV’s incubation period [102]. EBNA1 expression enhanced the proliferation and metastatic potential of EBV-negative NPC cells in immunodeficient mice [103]. The mechanism of EBNA1 in promoting cancer may include upregulating tumor angiogenic cytokines, disrupting p53 signaling, blocking DNA repair, inhibiting cell apoptosis and promoting epithelial-mesenchymal transformation [17,104,105]. EBNA1 can significantly upregulate the expression of various p53 inhibitory genes, such as mouse double minute (MDM)2, MDM4, histone deacetylase (HDAC) 1, etc. [106].

### 3.2. LMP1 and NPC

LMP1, a significant viral oncoprotein, is intimately associated with the transformation of epithelial cells. LMP1 can promote the development of tumors by damaging cell DNA and inducing epithelial mesenchymal transformation (EMT) [27,54,107,108]. LMP1 is predominantly present in precancerous lesions such as dysplasia and carcinoma in situ, suggesting its expression might be an early initiating event in NPC [109]. LMP1 has been proven to promote tumor invasion and metastasis by remodeling actin filaments and upregulating the expression of various matrix metalloproteins (MMPs) [110,111,112,113]. LMP1 can not only inhibit cell apoptosis, but also induce cancer stem/progenitor cells in nasopharyngeal epithelial cells [114,115]. Research indicates that LMP1 suppresses DNA damage binding protein expression via the PI3k/Akt signaling pathway, hindering DNA repair in epithelial cells and resulting in genomic instability in nasopharyngeal epithelial cells [116].

### 3.3. LMP2 and NPC

Expression of LMP2A in NPC cells induces cellular morphological alterations, leading to the loss of differentiation markers and cell anchorage, which subsequently enhances cellular proliferation capacity [117]. Furthermore, LMP2A stabilizes β-catenin via the protein kinase C-mediated inhibition of glycogen synthase kinase-3 (GSK-3), thereby promoting cell proliferation [118]. LMP2A mimics B-cell receptor (BCR) signaling, providing a robust BCR-like survival signal for EBV-infected B cells, thus protecting BCR-negative B cells from apoptosis and blocking the lysis reaction initiated by BCR stimulation [118]. This signaling mechanism may facilitate the evasion of host immune clearance by EBV-infected cells, thereby contributing to the onset and progression of NPC.

Like LMP1, LMP2 also has the ability to induce stem cells in NPC, and can induce epithelial mesenchymal transformation by upregulating various MMPs [114]. The N terminal cytoplasmic domain of LMP2 can be involved in the regulation of multiple signaling pathways, such as PI3K/Akt, RhoA, and MAPK/ERK, thus promoting the survival of epithelial cells [119,120]. LMP2A synergistically promotes epithelial cell dedifferentiation and maintenance of tumor stem cell characteristics through the ITAM motif (inhibiting differentiation) and PY motif (activating the Wnt/β–catenin pathway), thereby promoting EBV-associated epithelial cell carcinogenesis [121]. LMP2A also can promote the dedifferentiation of tumor cells and induce EMT by activating signaling pathways such as β–catenin, thereby enhancing the invasion and metastasis ability of tumors [122,123]. Research has shown that LMP2A drives the malignant progression of EBV-associated epithelial tumors by upregulating EMT transcription factors (such as Snail and ZEB1) and downregulating epithelial markers (such as E-cadherin) [124].

Additionally, CD21 serves as the primary receptor for EBV infection in B cells, mediating viral entry through its interaction with the EBV surface glycoprotein gp350/220 [54]. The expression pattern of CD21 in nasopharyngeal epithelial cells is complex. There is currently no conclusive evidence to suggest a direct interaction between LMP2 and CD21 in mediating the occurrence and development of NPC. However, both factors play a crucial role in the pathogenesis of EBV-induced NPC.

### 3.4. BZLF1 and NPC

As the core regulatory factor of the EBV lytic cycle, the ZEBRA (as known as BZLF1, Zta) protein exhibits multi-layered oncogenic mechanisms. Its molecular characteristics can be summarized as follows: (1) induction of genomic instability: triggering polyclonal B-cell activation through epigenetic reprogramming and mediating reactive oxygen species (ROS) burst leading to DNA double-strand breaks (e.g., IgH-MYC translocation) and cell cycle dysregulation; (2) transcriptional network remodeling: bidirectional regulation of host gene expression (upregulating pro-tumor factors/suppressing immune- and apoptosis-related genes); (3) dynamic protein interactions: forming microenvironment-dependent interaction networks with key proteins like p53. These mechanisms collectively constitute the theoretical basis for the “hit-and-run” oncogenic model, where even transient expression can induce persistent malignant transformation [125].

In terms of tissue-specific carcinogenesis, BZLF1 demonstrates significant pathological heterogeneity. In NPC, it activates the NF-κB/STAT3 pathway, upregulates pro-inflammatory factors (e.g., IL-6, IL-10) to induce a tumor-promoting microenvironment, and enhances metastatic potential through the EMT program [126]. Notably, its oncogenic effects show significant synergy with latent proteins (e.g., LMP1), jointly activating pro-survival pathways like AKT/mTOR and regulating cell cycle checkpoint proteins (e.g., p53), forming a vicious cycle of abnormal proliferation and anti-apoptosis [38].

### 3.5. EBERs

EBER is able to trigger an inflammatory response through the toll-like receptor (TLR) pathway, leading to elevated levels of tumor necrosis factor α (TNF-α), and synergies with LMP1 through a positive feedback loop mediated by NF-κB to amplify the inflammatory response in NPC [127]. EBER helps NPC cells evade recognition and attacks by the host immune system through a variety of mechanisms. For example, EBER was able to induce the expression of the immunosuppressive cytokine IL-10, thereby inhibiting the activity of cytotoxic T cells [128,129,130,131]. The role of EBER in nasopharyngeal cancer also includes promoting angiogenesis. Studies have shown that EBER promotes angiogenesis by upregulating the expression of VCAM-1 through TLR3 and RIG-I pathways [132]. In addition, EBER may promote tumor angiogenesis through other signaling pathways, such as PI3K/AKT and HIF-1α [133].

### 3.6. Immune Escape and EBV-Induced NPC

The host’s immune response to EBV infection plays a dual role in the development of NPC. On the one hand, the immune system attempts to eliminate viruses and prevent the development of malignant tumors. On the other hand, chronic immune stimulation of EBV may contribute to the carcinogenic process. In NPC, the characteristic of the immune microenvironment is the infiltration of immune cells, including T cells, B cells, macrophages, and natural killer (NK) cells. The role of these immune cells in tumor dynamics varies with their activation state and the presence of immunosuppressive elements, potentially leading to either anti-tumor effects or tumor promotion [134,135].

EBV infection can evade host immune attacks through various pathways. Typical mechanisms of immune evasion include interference with antigen presentation. For example, EBNA1 employs its Gly-Ala repeat domain to inhibit its own presentation on MHC class I molecules, thereby evading recognition by cytotoxic T cells [136]. BNLF2a suppresses the transporter associated with antigen processing (TAP), which reduces the binding of antigenic peptides to MHC class I molecules, thus limiting antigen presentation on the cell surface and decreasing the recognition of infected cells by CD8+ T cells [136]. LMP1 plays a pivotal role in the immune evasion mediated by EBV-infected cells. It induces the upregulation of chemokines such as MIP-1α and MIP-1β via CTAR1 and CTAR2, promoting the recruitment of T cells [137]. LMP1 can upregulate IL-10 expression, enhancing the function of regulatory T cells and reducing the infiltration of cytotoxic T cells [137]. Additionally, LMP1 regulates the PD-1/PD-L1 immune checkpoint through STAT3, AP-1, and NF-κB signaling pathways, further facilitating immune evasion. LMP1 can also upregulate the expression of PD-L1 by activating the NF-κB and JAK/STAT signaling pathways [138,139]. Experimental data show that LMP1 can increase the expression level of PD-L1 by 4–6 times and significantly inhibit the killing function of CD8+ T cells. In addition, LMP1 can induce the expression of Galectin-9, which promotes T cell apoptosis by binding to TIM-3 receptors [140]. Another important protein, LMP2A, promotes tumor cell immune escape through the PI3K/Akt/mTOR pathway, while inhibiting the expression of MHC class I molecules and reducing tumor cell immunogenicity [141,142]. Collectively, these mechanisms enable EBV-infected cells to evade host immune surveillance, thereby supporting their survival and proliferation and contributing to the development of NPC.

The high expression of EBER in EBV-related NPC patients can also promote immune escape by inhibiting the gene activity stimulated by interferon [25]. LMP1 can promote the secretion of chemokine CCL20 through the NF-κB signaling pathway and recruit Treg cells to migrate towards tumor tissue to induce immune suppression [143]. LMP2 can also inhibit type I IFN response in epithelial cells by suppressing the activity of IFN-secreting genes [144]. In addition, studies have found that EBV encoded miRNAs are abnormally expressed in NPC, which may be closely related to immune escape in the body [145,146,147]. BART miRNAs encoded by EBV are abundantly expressed in NPC, facilitating immune evasion by suppressing MHC-I expression, and regulating various biological behaviors of cells, such as proliferation, apoptosis, migration, and invasion, by targeting multiple host genes [148,149].

In addition, NPC-derived small extracellular vesicles (NPCSEVs) also have an immunosuppressive impact on the tumor microenvironment. NPCSEVs can significantly inhibit CD8+ T cell function and promote Treg expansion by delivering EBV-encoded BART miRNAs (such as BART1-3p) and immunosuppressive proteins (such as LMP1, Galectin-9, PD-L1), thereby mediating immune escape. Experimental results have shown that NPCSEVs can downregulate key molecules in the TCR signaling pathway, such as CD3 Zeta and ZAP70, while inducing polarization of M2 macrophages and activation of CAFs, synergistically constructing an immunosuppressive microenvironment [150,151]. Another study revealed that NPC exosomes (NPC Exo) significantly recruited and activated Tregs through a CCL20-dependent mechanism (increased 3.3-fold, *p* < 0.001), while inducing CD4+ T cells to transform into immunosuppressive Tregs (FOXP3+/GZMB+), ultimately promoting immune escape by secreting IL-10/TGF-β1 [152]. In addition, FasL/TRAIL carried by NPCSEVs can directly induce T cell apoptosis, while EBV miRNAs further stabilize the immunosuppressive phenotype through epigenetic regulation [153]. These findings provide new strategies for targeted combination immunotherapy of NPCSEVs.

### 3.7. Epigenetic Changes and EBV-Induced NPC

Epigenetic changes may also be one of the reasons why EBV leads to tumorigenesis [154]. CpG hypermethylation serves as a prevalent mechanism for silencing tumor-suppressor genes. In host cells, the transition from methylation to CpG island hypermethylation has been noted in EBV-related Burkitt lymphoma, NPC, and gastric cancer [155]. Remarkably, this epigenetic alteration triggered by EBV persists in infected cells even subsequent to the eradication of EBV fragments, suggesting a “hit and run” mechanism of EBV infection [156]. Although host genome methylation induced by EBV infection may include both specific and non-specific methylation, hypermethylation of CpG island, a promoter region of tumor-suppressor genes, certainly provides a growth advantage to EBV-infected cells [157,158]. DNA methyltransferase activated by LMP1 activation plays an important role in promoting and maintaining viral and host genomic DNA methylation [157]. Furthermore, EBV-encoded miRNAs play crucial roles in promoting NPC metastasis. For example, BART1-3p targets PTEN, leading to activation of PI3K/Akt and MAPK/ERK pathways [77]. BART1-3p expression has been shown to correlate with metastatic potential and poor prognosis in NPC patients.

#### EBV and NPC Stem Cells

NPC stem cell characteristics are pivotal in sustaining EBV latent infection and the malignant transformation of nasopharyngeal epithelial cells. CD44^+^SOX2^+^ spheroid stem cells isolated from the EBV-positive NPC cell line C666-1 significantly enhance tumorigenic potential and induce chemotherapy resistance [159]. The EBV copy number, EBER, and LMP1 expression are markedly elevated in NPC spheroid stem cells, indicating a close relationship between NPC stem cells and EBV latent infection [159]. Overexpression of LMP1 and LMP2 promotes EMT and induces the formation of cancer stem cells (CSCs) by upregulating the expression of stem cell surface markers [115,160]. Additionally, EBV-activated hedgehog and PI3K/Akt signaling pathways also play a pivotal role in the induction of NPC CSCs [114].

## 4. How to Choose Effective Vaccine Antigens

In the development of vaccines targeting the EBV, selecting effective antigens is crucial to ensuring the immunogenicity and therapeutic efficacy of the vaccine. The EBV lifecycle is complex, involving multiple latent and lytic proteins, which exhibit distinct expression patterns across different EBV-associated diseases. Therefore, the selection of antigens should comprehensively consider factors such as the expression, immunogenicity, functional importance, and conservation of EBV antigens. Proteins expressed during EBV latent infection, such as EBNA1, LMP1, and LMP2A, are key targets for vaccine design [161]. These proteins are persistently expressed in EBV-associated tumors (e.g., NPC and Hodgkin’s lymphoma) and are closely related to tumor cell survival and proliferation [120,162]. For instance, a pan-HLA class II-restricted EBV peptide cocktail (derived from LMP1/LMP2/EBNA1) targeting EBV latency II-associated malignancies (such as Hodgkin’s lymphoma and NPC) has been shown to activate CD4+ T cell responses, offering a potential vaccine strategy against EBV-associated malignant tumors. Lytic proteins, such as BZLF1 and BALF4, play a critical role in EBV replication and spread, and vaccines targeting these antigens can prevent viral dissemination and reinfection [163]. Studies have shown that LMP1 and LMP2A can induce strong CD8+ T cell responses, while gp350 can elicit the production of neutralizing antibodies [164,165]. To ensure broad-spectrum protection, it is essential to select antigens that are highly conserved across different EBV strains. For example, EBNA1 is highly conserved in nearly all EBV strains, making it an ideal target [90,166]. Additionally, EBNA1 is crucial for maintaining EBV genome replication and distribution in host cells, while LMP1 and LMP2A play key roles in cell signaling and immune evasion [90,167]. Therefore, adopting a multivalent vaccine strategy that targets multiple EBV antigens may be more effective than a single-antigen vaccine [161,168].

### 4.1. EBV Vaccine Vector and Delivery Strategy

The efficacy of EBV vaccines heavily depends on the choice of delivery systems, which influence antigen presentation, immune activation, and long-term protection. Viral vectors (e.g., MVA and adenovirus) effectively deliver antigens to antigen-presenting cells APCs, as demonstrated by MVA-encoded EBNA1/LMP2 vaccines inducing robust CD8+ T-cell responses [164,169]. Non-viral platforms like LNPs—successful in COVID-19 mRNA vaccines—offer safety and multi-antigen delivery potential for EBV [170,171]. Additionally, autologous DC vaccines loaded with EBV antigens elicit strong T-cell immunity. Current research focuses on enhancing APC targeting (e.g., DEC-205 antibodies) and combining adjuvants (e.g., TLR agonists) to optimize immunogenicity and durable protection [172,173,174,175]. Overall, the optimal EBV vaccine delivery strategy should balance effective antigen presentation, safety, and induction of persistent immunity.

### 4.2. EBV Vaccine and Immune Adjuvant

Immune adjuvants can to some extent enhance the immunogenicity of EBV vaccines, thereby triggering strong and long-lasting immune responses. The commonly used immune adjuvants currently include TLR agonists, such as TLR4 agonist monophosphoryl lipid A (MPL) and TLR9 agonist CpG oligodeoxynucleotides, which can activate DC and enhance T cell initiation [175,176,177,178]. Another type is based on nanoparticle-delivery systems, such as LNPs or polymer nanoparticles, which can protect antigens from degradation and promote their uptake by APCs, thereby improving antigen presentation and immune activation [170]. Molecular adjuvants such as cytokines (such as IL-12, GM-CSF) could also be co-administered with vaccines to regulate the immune microenvironment and promote Th1 or cytotoxic T lymphocyte (CTL) responses [179]. In addition, optimizing antigen design, such as using multivalent antigens or fusion proteins, can enhance the breadth and efficacy of immune responses [168]. Combining these strategies with advanced delivery platforms such as viral vectors or mRNA vaccines can further enhance immunogenicity by ensuring effective antigen expression and presentation [180]. Overall, the integration of adjuvants, delivery systems, and optimized antigen design is crucial for the development of highly immunogenic EB virus vaccines.

## 5. Targeting EBV Vaccines for the Prevention and Treatment of NPC

The concept of using vaccines to prevent EBV-related malignancies or reduce their incidence was first proposed by Epstein in 1976 [181]. To date, based on design objectives and mechanisms of action, vaccines targeting EBV can be categorized into two main types: preventive vaccines and therapeutic vaccines. Because the proteins expressed in different incubation periods of EBV are different, which is also related to the occurrence and development of different types of tumors, according to the characteristics of EBV, the specific tumor vaccine can be more targeted, and two or more proteins can be integrated. We aim to improve the probability of vaccine treatment of EBV-mediated related diseases and/or tumors [162,182]. Common EBV vaccine platforms include viral vector vaccines, DC vaccines, nanoparticle vaccines, and mRNA vaccines, which have gained prominence during the COVID-19 pandemic. Although clinical trials have demonstrated promising potential for EBV-targeted vaccines in treating NPC, no such vaccines have been approved for market release [183]. In the long term, EBV vaccines remain the most cost-effective and efficient method for preventing and treating EBV-associated cancers, IM, and autoimmune diseases [184,185].

## 6. Preventive Vaccines

The primary applications of preventive EBV vaccines include preventing IM, reducing the incidence of EBV-related diseases, inducing mucosal immune responses, and lowering the risk of EBV-associated tumors by preventing EBV infection. The ideal timing for vaccination is pre-adolescence (10–12 years old) or EBV seronegative adolescents or adults [1]. The current summary table of clinical studies on preventive EBV vaccines is shown in Table 1.

### 6.1. Targeted-gp350

Early research on EBV vaccines primarily focused on gp350, the most abundant envelope protein of EBV, due to its association with neutralizing antibodies produced by most patients [62,186]. gp350 is a major membrane glycoprotein and a key component for viral entry into host cells. Studies have shown that recombinant gp350 protein vaccines induce neutralizing antibodies in 70% of EBV-infected individuals and reduce the incidence of IM by 78%. However, this vaccine has limited efficacy in preventing EBV infection [187], likely because gp350 is not essential for EBV to infect B cells [188,189].

### 6.2. Targeted-gB

gB is an EBV fusion protein that mediates the fusion of the virus with the host cell membrane, making it an ideal vaccine target [190,191]. The gB-I53-50 NP nanoparticle vaccine developed by researchers from Sun Yat-sen University and Southern University of Science and Technology was designed through computer-aided methods to display gB proteins on the surface of nanoparticles, significantly enhancing antigen immunogenicity and stability. Animal studies have shown that this vaccine efficiently induces EBV-neutralizing antibodies, inhibits infection of epithelial and B cells, and enhances T-cell activation. Additionally, this vaccine demonstrates significant protective effects in humanized mouse models, preventing EBV infection and lymphoma [192].

### 6.3. Nanoparticle Vaccines

Nanoparticle vaccine technology improves vaccine efficacy by optimizing antigen presentation and immune activation. For example, the aforementioned gB nanoparticle vaccine has been structurally optimized using cryo-electron microscopy, demonstrating excellent antigenicity and stability. This vaccine not only induces highly effective neutralizing antibodies but also provides long-lasting protection, laying a foundation for the development of next-generation preventive EBV vaccines [192]. Li et al. developed a self-assembling nanoparticle-based vaccine that significantly improved vaccine efficacy by optimizing antigen presentation and immune activation [193]. The L350-ferritin nanoparticle vaccine induced highly efficient neutralizing antibodies and demonstrated higher immunogenicity and long-lasting protective effects in immunized mice. Compared to monomeric antigens, the L350-ferritin nanoparticle vaccine induced higher titers of neutralizing antibodies and significantly increased memory B cell generation, indicating its ability to establish long-term immune memory [193].

### 6.4. Virus-like Particle Vaccines

A key challenge in developing EBV virus-like particle (VLP) vaccines is the exclusion of potential oncoproteins (e.g., EBNA2, LMP1, BZLF1) from vaccine components due to their cancer risks. In 2020, Escalante et al. found that a pentavalent EBV glycoprotein (gp350, gB, gp42, gH, and gL) VLP formulation induced a more efficient humoral immune response in rabbits compared to soluble gp350 extracellular domains and provided better protection against EBV infection of B and epithelial cells [178]. However, the possibility of EBV-DNA repackaging remains a significant safety concern for EBV-derived VLPs. Some studies have attempted to use Newcastle disease virus (NDV) to prepare VLPs containing EBV gp350/220, gH/gL, EBNA1, gB, or LMP2, but vaccine efficacy has been inconsistent and inferior to ultraviolet-inactivated EBV [194,195].

Despite significant progress in preventive vaccine research, several challenges remain. For instance, the protective effect of gp350 vaccines in humans is limited, and the safety and efficacy of novel nanoparticle vaccines require further verification through clinical trials. Additionally, vaccine development must consider variations in immune responses among different populations and potential side effects.

**Table 1:** Clinical vaccine studies on prophylaxis vaccines against EBV.

Vaccine	Population	Dose	Year	Number	Phase	Primary Outcome	Adverse Reactions	NCT Number	Reference
Recombinant gp350 vaccine	EBV-seronegative, healthy, young adult volunteers	Each dose of vaccine contained 50 g of gp350 and AS04, intramuscularly into the deltoid at 0, 1, and 5 months	16–25 years	181	II	Significant prevention of EBV-induced IM. Not effective for asymptomatic EBV infection.	Well-tolerated and no severe adverse events.	NCT00430534	[165]
EBNA3 peptide	Healthy, EBV-seronegative and HLA B * 0801-positive volunteers	Volunteers were given 0.5 mL of vaccine per injection subcutaneously into the thigh	18–50 years	14	I	Inducing CD8+ T immune response, preventable infectious mononucleosis.	Well-tolerated and the main side effects are mild to moderate injection site reactions.	N/A	[196]
Recombinant EBV gp350 Vaccine purified from CHO cells	Healthy volunteers	Inject 50 g gp350 intramuscularly at 0, 1, and 6 months	18–24 years	148	I/II	The gp350 vaccine formulations were immunogenic and induced gp350-specific antibody responses.	Safe and well-tolerated.	N/A	[187]
gp350 EBV vaccine	Chronic kidney disease (CKD)	Immunization with 12.5 µg or 50 µg gp350	1.4–17.6 years	16	I	Most patients displayed nAb responses.	Safe and well-tolerated; only two patients had systemic reactions.	N/A	[197]
Recombinant vaccinia virus expressing the major membrane antigen (gp350)	a. EBV-positive and vaccinia-exposed adults. b. 8 to 9 year old juveniles. c. 1 to 3 year old infants	Immunization with 10 [7] pfu/mL or 108 pfu/mL of vaccinia virus expressing gp220-340	Adults; 8–9 years; 1–3 years	36	I	Adults who have previously been exposed to the smallpox virus can impair their immune response to the vaccine. Adolescents and infants who have not been vaccinated have a strong overall response to the vaccine.	Safe and well-tolerated.	N/A	[198]

CHO: Chinese hamster ovary; EBV: Epstein–Barr virus; HLA: human leukocyte antigen; IM: infectious mononucleosis. N/A: not applicable. The symbol “*” is used in HLA gene naming to separate gene names from allele numbers.

## 7. Therapeutic Vaccines

### 7.1. Multi-Epitope Recombinant Vaccines

Therapeutic vaccines targeting EBV are designed to treat EBV-related diseases such as NPC, Hodgkin’s lymphoma, and post-transplant lymphoproliferative disorder by activating the immune system to eliminate or suppress EBV-infected tumor cells. Key targets include LMP1, LMP2, and EBNA1. In addition to single glycoprotein vaccines used in preventive strategies, researchers have explored multi-epitope recombinant vaccines that combine multiple EBV glycoproteins (e.g., gB, gH/gL, and gp42) [186]. These vaccines induce specific T-cell immune responses by fusing immunogenic epitopes from latent EBV proteins such as LMP1, LMP2A, and EBNA1 [1,199]. For example, a multi-epitope vaccine combining LMP1 and LMP2 epitopes with a truncated EBNA1 gene has successfully induced specific T-cell responses in mouse models and demonstrated significant anti-tumor effects [200]. Studies suggest that a combination of gH/gL, gB, and gp350 may be an ideal preventive vaccine strategy, eliciting robust neutralizing antibodies and CD4+/CD8+ T-cell responses, thereby enhancing vaccine efficacy [28,201].

### 7.2. DC Vaccines

Early therapeutic vaccines for EBV were based on DCs [20]. Autologous DC vaccines, a personalized immunotherapy approach, can be loaded with EBV antigens in vitro and have demonstrated promising efficacy in inducing robust T-cell responses against EBV-related malignancies [172,202]. For instance, KSD-101 is an autologous dendritic cell (DC) -based vaccine targeting EBV-associated malignancies. In a study involving five evaluable patients with EBV-associated hematological malignancies, the complete response rate (CR) reached 100%. Following treatment, the levels of various immune cells in peripheral blood significantly increased, with the proportion of EBV-specific CTLs rising from an average of 0.30% at baseline to 2.47% at 12 weeks post-treatment. No dose-limiting toxicity (DLT) or maximum tolerated dose (MTD) was observed (NCT06097793) [203]. Lin et al.’s study demonstrated that all 16 NPC patients who received autologous DC vaccines stimulated by LMP2 antigens generated EBV-specific CD8+ T-cell responses, with two patients showing significant tumor regression [204] (Table 2).

In order to enhance the targeting and uptake of APCs, strategies such as coupling antigens to antibodies targeting DC surface receptors (such as DEC-205) or using pH-sensitive nanoparticles for in vivo escape are being studied [173,174]. Combining these delivery systems with immune adjuvants such as TLR agonists can further enhance innate and adaptive immune responses [175]. Overall, the optimal EBV vaccine-delivery strategy should balance effective antigen presentation, safety, and induction of persistent immunity. However, DC vaccines provide limited antigenic epitopes and are costly to produce, restricting their clinical application [20].

### 7.3. Recombinant Viral Vector Vaccines

The selection of vaccine carriers and delivery strategies is crucial for the efficacy of EBV vaccines, as they directly affect antigen presentation, immune activation, and the persistence of protective responses. Recombinant virus vector vaccines offer a broad range of antigenic epitopes. Viral vectors, such as modified Ankara vaccine (MVA) and adenovirus, are widely used due to their high immunogenicity and ability to effectively deliver EBV antigens to APCs [164,169]. For example, a recombinant adenovirus vector vaccine delivering the LMP2 antigen can dose-dependently increase the proportion of LMP2-specific CD3+/CD4+ T lymphocytes in the peripheral blood of NPC patients [205]. Another virus vector vaccine expressing a fusion protein of EBNA1 and LMP2 (MVA-EL) induces specific CD4+ and CD8+ T-cell responses against EBNA1 and LMP2 [206]. A phase I clinical trial of MVA-EL showed that among 18 NPC patients receiving intradermal immunization, 15 patients exhibited a twofold increase in T-cell responses to EBNA1 and LMP2 antigens [169] (Table 2). Although these vaccines can deliver multiple antigenic epitopes simultaneously, the immune response to the viral vector can reduce therapeutic efficacy. Additionally, there is a risk of integration into the host genome, potentially leading to gene mutations. Therefore, their clinical application is somewhat limited. In addition to these vaccines, this article also summarizes early clinical research on EBV-targeted vaccines for the treatment of NPC (Table 1).

### 7.4. mRNA Vaccines Targeting EBV

#### 7.4.1. Mechanism and Advantages of mRNA Vaccines

**Figure 2:**
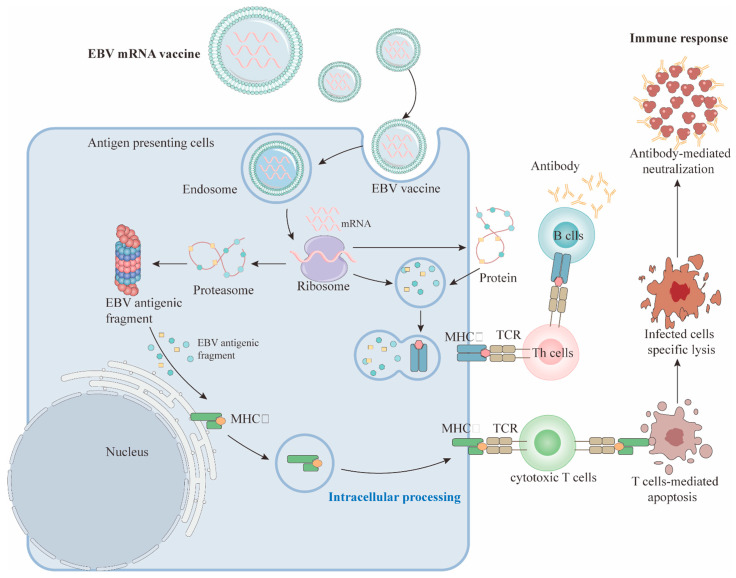
The anti-tumor mechanism of EBV mRNA vaccine. mRNA vaccines are typically encapsulated in lipid nanoparticles (LNPs) to protect the mRNA from degradation and facilitate its entry into APCs (such as DCs). After uptake and translation by APCs, the antigen proteins are degraded into peptides and presented to CD8+ and CD4+ T cells via MHC class I or II molecules. Antigen proteins may also be secreted extracellularly and recognized by B cells, thereby activating them and generating specific antibodies [219,220,221].

As one of the non-viral vector vaccines, mRNA vaccines include carriers such as LNP and nanoparticles, and have advantages such as safety, stability, and the ability to co-deliver antigens and adjuvants. In particular, the success of the COVID-19 mRNA vaccine proves that LNP (lipid nanoparticles) has excellent delivery effects, so researchers have also begun to explore how to use the EBV mRNA vaccine to encode multiple EBV antigens [170,171]. mRNA vaccines deliver antigen-encoding mRNA molecules into cells, where the cellular translation machinery synthesizes the target antigen proteins. These proteins subsequently activate the adaptive immune response, enabling the body to recognize and eliminate the corresponding pathogen. Upon injection, mRNA vaccines are taken up by APCs, such as DCs. The mRNA is then released from lysosomes and translated by ribosomes into specific antigen proteins. These antigens are presented via MHC-I and MHC-II molecules on DCs, triggering CD8+ and CD4+ T cell responses, ultimately leading to tumor elimination [207] (Figure 2). Additionally, after being internalized by APCs, mRNA vaccines can be recognized by sensors such as TLR3, TLR7/8 in the endoplasmic reticulum and RIG-I, NOD2, LGP2, and MDA5 in the cytoplasm, inducing type I interferon (IFN-I) responses and activating innate immunity.

The advantages of mRNA vaccines include the following [208]: (1) mRNA vaccines can precisely encode multiple antigens tailored to the characteristics of different diseases, eliciting both innate and adaptive immune responses [209,210]. (2) Leveraging efficient in vitro transcription technology, mRNA vaccines enable rapid design, development, and large-scale production [211,212,213,214,215,216]. (3) Absence of live viruses and no integration into the host genome, avoiding the risk of insertional mutagenesis [214,215,216]. (4) Efficient cellular entry and immune activation through delivery systems such as lipid nanoparticles (LNPs) [217,218]. For example, Moderna’s mRNA-1189 vaccine targets EBV envelope glycoproteins and is delivered via LNPs, effectively activating the immune system (NCT05164094).

#### 7.4.2. Progress of mRNA Vaccines Targeting EBV in NPC

mRNA vaccines represent a significant breakthrough in the field of therapeutic vaccines. The successful development of mRNA vaccines against SARS-CoV-2 has also accelerated advancements in the synthetic mRNA vaccine industry [201,222]. The first candidate mRNA vaccine for preventing EBV infection, mRNA-1189, produced by Moderna, entered Phase I clinical trials in 2022 (NCT05164094). This trial aims to evaluate the safety and tolerability of three different doses of mRNA-1189 in healthy adults aged 18–30 years [223]. The candidate vaccine contains five mRNAs encoding EBV envelope glycoproteins (gp320, gH, gL, gp42, and gp220), expressed in their natural membrane-bound conformations, designed to elicit a broad immune response and reduce the incidence of IM. The results indicated that the treatment regimen was well tolerated, with no serious adverse events reported, while common adverse reactions primarily included mild pain and redness at the injection site [223].

But, outside of infectious mononucleosis, the clinical research on vaccines targeting EBV for the treatment of NPC remains limited. Recently, Guo et al. developed a liposome-based LMP2-mRNA vaccine (LPX-mLMP2) targeting NPC. In vitro and in vivo experiments showed that this vaccine stimulates antigen-specific T cell responses by promoting DC maturation and significantly inhibits the growth of LMP2-expressing tumors in vivo [224]. Subsequently, Zeng et al. developed three mRNA vaccines encoding truncated forms of potential EBV proteins: Trunc-LMP2A, Trunc-EBNA1, and Trunc-EBNA3A. These vaccines effectively activated cellular and humoral immunity in animal models, significantly inhibiting tumor progression and prolonging the survival time of tumor-bearing mice [225]. The study indicated that using truncated forms of antigens enhances specific immune activation [225]. WGc-043 injection, developed by WestGene, is an mRNA vaccine targeting EBV-related malignancies and has received clinical trial approval in both China and the United States. This vaccine employs artificial intelligence to screen for broad-spectrum safe protein sequences and introduces immune enhancers (IEs) to activate the patient’s own anti-tumor immune response, generating CTLs, antigen-specific antibodies, and memory T cells. The preliminary results of the first phase clinical study initiated by researchers on WGc-043 have been published, indicating that the vaccine has good safety (NCT05714748) [226]. The study recruited 12 EBV-positive recurrent or metastatic NPC patients aged ≥ 18 years, who were vaccinated with WGc-043 vaccines at doses of 25 μg, 50 μg, and 100 μg, respectively. The results showed a disease-control rate (DCR) of 66.67% and an objective response rate (ORR) of 16.67%. In addition, 66.7% of patients developed strong specific immune responses to EBV-associated antigens, and 91.7% of patients had significantly reduced plasma EBV DNA levels [226]. Nonetheless, research on therapeutic mRNA vaccines for EBV-induced NPC remains limited, and no such vaccines have been approved for marketing.

**Table 2:** Clinical studies on therapeutic vaccines against EBV in NPC.

Vaccine	Disease	Dose	Number	Phase	Primary Outcome	Reference
Recombinant vaccinia virus, MVA-EMVA EBNA1/LMP2 vaccine	NPC	Received three intradermal MVA-EL vaccinations at three weekly intervals, using five escalating dose levels between 5 × 10^7^ and 5 × 10^8^ plaque-forming unit (pfu)	18	I	T cell response was increased in 15 patients. The vaccine was immunogenicity and safety.	NCT01256853 [169]
LMP2 vaccine	NPC	N/A.	99	I	Not posted	NCT00078494 [223]
MVA EBNA1/LMP2 vaccine	NPC	3-weekly intervals at dose levels between 5 × 10^7^ and 5 × 10^8^ plaque-forming units (pfu).	16	I	The vaccine was safety, 8/14 patients (7/14, EBNA1; 6/14, LMP2) enhanced immunity after inoculation with at least one antigen, differentiation and functional diversification of EBNA1 and LMP2-specific CD4+ and CD8+T cells were induced.	NCT01800071 [224,225]
AdE1-LMP poly vaccine	NPC	Received 3 to 8 infusions of 2 × 10^7^ to 3 × 10^7^ AdE1-LMPpoly–expanded T cells per infusion.	24	I	T cells specific to EBV were successfully expanded in 16 patients: 10 patients received SD, 4 patients received PD.	ACTRN12609000675224
Adenovirus ΔLMP1–LMP2 Transduction DC vaccine	NPC	i.d. biweekly for up to five doses.	16	I	There was no increase in peripheral LMP1/2 specific T cells.	[227]
EBV-specific HLA-A2- Restricted DC vaccine	NPC	Intradermal injection near inguinal lymph nodes, 1 × 10^7^ each time, for a total of 4 times.	16	I	9 patients responded to the LMP2A peptide, and serum EBV-DNA levels were significantly reduced.	[2]
EBV-LMP2(rAd5-EBV-LMP2)	NPC	Three dose level groups (2 × 10^9^, 2 × 10^10^, 2 × 10^11^). The rAd5-EBV-LMP2 vaccines were intramuscularly injected for four times within 28 d (D0, D7, D14, D28).	24	I	Peripheral CD3+, CD4+ T was significantly increased, with safety and no vaccine efficacy.	[205]
LMP2-DCs vaccine	NPC	2 × 10^5^ LMP2-DCs by intradermal injection at week 0 and after the second and fourth weeks.	29	I	Boosted responses to LMP2 peptide sub-pools were observed in 18 of the 29 patients with NPC; five-year survival rate of 94.4% in responders and 45.5% in non-responders.	[3]
CD137L-DC-EBV-VAX	NPC	5–50 × 10^6^ cells were intradermal near the inguinal region at 2 weekly intervals.	12	I	CD137L-DC-EBV-VAX can induce an anti-EBV and anti-NPC immune response.	NCT03282617 [228]
EBV peptide-pulsed DCs vaccine	NPC	Inguinal lymph node injection once a week, a total of 4 times.	16	I	Epitope-specific CD8+ T-cell responses were elicited or boosted; without serious side effects.	[204]
EBV mRNA vaccine (WGc-043)	NPC	The patients received a dose of 25 μg, 50 μg or 100 μg.	12	I	Disease control rate (DCR) of 66.67% and an objective response rate (ORR) of 16.67%.	NCT05714748 [226]

DC: dendritic cell; EBV: Epstein–Barr virus; EBNA1: EBV nuclear antigen 1; LMP: latent membrane protein; MVA: modified vaccinia Ankara; NPC: nasopharyngeal carcinoma; N/A: not applicable.

## 8. Discussion and Outlook

Although advancements have been made in preventing and treating EBV-associated NPC, numerous challenges remain. On the one hand, EBV usually does not cause obvious clinical symptoms during the first infection, which increases the difficulty of its prevention. On the other hand, due to the lack of specific tumor markers for the occurrence and development of NPC, and the complex tumor immune microenvironment, the development and efficacy verification of EBV therapeutic vaccines also pose certain difficulties. Firstly, the lifecycle of EBV is complex, involving multiple latent and lytic proteins, which have varying expression patterns in different types of EBV-related tumors. Therefore, it is crucial to develop vaccine strategies that can simultaneously target multiple EBV antigens. Multivalent vaccines or combination vaccines may provide more comprehensive immune protection and reduce the possibility of virus escape. Secondly, the tumor microenvironment of EBV-related NPCs has a high degree of immune suppression, which limits the effectiveness of vaccine-induced immune responses. Future research endeavors can concentrate on devising strategies to counteract the immunosuppressive microenvironment, including the utilization of combinations of immune checkpoint inhibitors (like PD-1/PD-L1 antibodies) and vaccines in order to bolster anti-tumor immune responses. In addition, the application of nanotechnology and novel adjuvants may also enhance the immunogenicity and targeting of vaccines, thereby improving therapeutic efficacy.

Another important challenge is the genetic diversity and antigenic variation of EBV. There may be significant genetic differences among EBV strains in different geographical regions and populations, which may affect the broad-spectrum and effectiveness of vaccines. Therefore, future vaccine development should consider the global genetic diversity of EBV and design universal vaccines that can cover multiple EBV strains. The approval and marketing of mRNA vaccines for COVID-19 prevention have significantly advanced their application in cancer treatment. Because of its ability to encode multiple neoantigens, mRNA has become a leader in the development of personalized vaccines. mRNA personalized vaccines delivered by LNPs have achieved remarkable anti-cancer effects in a variety of solid tumors, thus opening a new era of therapeutic cancer vaccines. Therefore, the development of mRNA cancer vaccines targeting EBV may provide a new therapeutic strategy for the treatment of EBV-associated diseases.

Compared to DC vaccines and recombinant viral vector vaccines, nucleic acid vaccines offer several advantages. They can simultaneously deliver multiple tumor-specific antigens, significantly reducing the risk of vaccine resistance. Additionally, nucleic acid vaccines minimize the limitations imposed by human leukocyte antigen (HLA) diversity, thereby eliciting a broader range of antigen-specific immune responses [227,229]. Given the critical role of EBV in the pathogenesis of NPC, mRNA vaccines targeting EBV not only hold promise for preventing EBV infection but also serve as therapeutic vaccines for NPC patients. mRNA vaccines offer the flexibility to encode a wide range of antigens tailored to the specific characteristics of various diseases. Unlike DNA vaccines, mRNA vaccines eliminate the risk of insertional mutagenesis in the host genome and allow for precise control over the expression levels of the target antigen [214,215,216]. From a commercial perspective, mRNA vaccines can be rapidly developed and produced at scale using a cell-free system, thanks to the highly efficient in vitro transcription process, which is both time-saving and cost-effective [211,212,213,214,215,216]. However, mRNA vaccines still have some drawbacks, including poor stability (requiring low-temperature storage), the potential for transient immune-related adverse reactions (such as fever and fatigue), and the possibility of tumor microenvironment immunosuppression limiting efficacy [230,231]. In summary, mRNA vaccines remain an attractive class of nucleic acid vaccines for EBV-targeted prevention and treatment of NPC.

However, mRNA vaccines also face certain limitations. For example, mRNA molecules are susceptible to degradation by RNases in vitro, have a short half-life, and require specialized delivery systems such as lipid nanoparticles (LNPs) to protect them from degradation. mRNA vaccines typically need to be stored and transported at low temperatures; for example, Moderna’s mRNA-1273 vaccine requires storage at approximately −20 °C (NCT04470427). Moreover, the protective efficacy of mRNA vaccines may diminish over time, particularly in the face of viral mutations. Despite their significant potential in EBV-related diseases, further research is necessary to optimize vaccine design and delivery systems. Additionally, mRNA vaccines targeting EBV need to undergo clinical trials in larger and more diverse populations to verify their long-term safety and efficacy. However, addressing the inherent immunogenicity and instability of mRNA vaccines, we can choose to modify the 5′ cap structure [232], optimize the untranslated region [228], optimize the codon [233,234], modify the polyA [233,235,236], and modify the nucleotide [215,237,238,239,240,241,242,243] and other technical means to achieve a breakthrough in mRNA vaccine research and development. Future efforts should focus on optimizing mRNA structure and developing novel delivery carriers, as these are crucial for enhancing mRNA translation efficiency and anti-cancer efficacy.

To enhance the efficacy and applicability of EBV-targeted vaccines, future research and development should focus on the following areas: (1) Enhancing immunogenicity by incorporating multiple EBV antigen epitopes. Personalized mRNA vaccines can be developed based on individual genetic backgrounds and EBV infection characteristics, leveraging high-throughput sequencing and bioinformatics analysis. (2) Optimizing the distribution and metabolism of LNPs, the most commonly used mRNA delivery system. Adjusting LNP composition and surface modifications can improve tumor tissue targeting while reducing liver accumulation. Exploring delivery systems based on biodegradable polymers or other nanomaterials could further enhance mRNA stability and delivery efficiency. (3) Boosting immunogenicity through the addition of immune adjuvants such as TLR agonists or cytokines. For example, TLR5 agonists have been shown to significantly enhance the immune response of mRNA vaccines [244]. (4) Combining mRNA vaccines with other immunotherapies, such as immune checkpoint inhibitors, to improve therapeutic outcomes. (5) Improving mRNA stability and reducing immunogenicity via chemical modifications like pseudouridine substitution, and optimizing mRNA sequence design to enhance translation efficiency and stability.

## 9. Conclusions

Substantial progress has been made in developing EBV vaccines for NPC prevention and therapeutic intervention. Current strategies—including gp350-focused prophylactic vaccines and latency antigen-targeted therapeutic vaccines—demonstrate promising immunogenicity in preclinical and early clinical trials. However, challenges such as viral antigenic variability, immune evasion mechanisms, and the complexity of EBV latency phases necessitate further optimization of vaccine design. Future success will likely depend on combinatorial approaches integrating novel adjuvants, delivery platforms (e.g., mRNA or viral vectors), and checkpoint inhibitors to enhance durable immunity.

While EBV vaccine research holds transformative potential for NPC management, critical questions remain. Given the geographic skew of NPC endemicity, should future trials prioritize region-tailored vaccines based on EBV strain prevalence or host genetic factors (e.g., HLA subtypes)? Could EBV vaccines synergize with emerging therapies like EBV-specific TCR-T cells, and how should sequential or combined regimens be evaluated? For accelerated clinical translation, what biomarkers (e.g., EBV DNA load, T-cell clonality) best predict long-term vaccine efficacy in prevention vs. treatment settings? Addressing these questions will require interdisciplinary collaboration among virologists, immunologists, and oncologists to bridge the gap between bench research and global NPC burden reduction.

## Abbreviations

BMRF2: Bone Morphogenetic Protein Receptor 2; BART: BamHI-A region rightward transcript; BCR: B-cell receptor; BZLF1: BamHI Z Leftward Reading Frame 1; EBV: Epstein–Barr virus; EBER: EBV-encoded small RNA; EBNA: EBV nuclear antigen; EA: Early antigen; EMT: Epithelial mesenchymal transformation; EphA2: Ephrin type-A receptor 2; EBD: Ephrin-binding domain; FNR: Fibronectin III type repeats; HDAC: Histone deacetylase; GSK-3: Glycogen synthase kinase-3; LMP: Latent membrane protein; MDM:Mouse double minute; MMPs: Matrix metalloproteins; NPC: Nasopharyngeal carcinoma; NMHC-IIA: Non-musclemyosin heavy chain IIA; NRP1: Neuropilin-1; PI3K: Phosphatidylinositide 3-kinases; PKB: Protein kinase B; RIG-I: Retinoic Acid-Inducible Gene I TLR3:Toll-like receptor 3; ROS: Reactive oxygen species; TNF-α:tumor necrosis factor α; VCA: Virus capsid antigen; VCAM: vascular cell adhesion molecule.
